# Investigation of CO_2_ Photoreduction in an Annular Fluidized Bed Photoreactor by MP-PIC Simulation

**DOI:** 10.1021/acs.iecr.1c04035

**Published:** 2022-02-21

**Authors:** Xuesong Lu, Jeannie Z. Y. Tan, M. Mercedes Maroto-Valer

**Affiliations:** Research Centre for Carbon Solutions (RCCS), School of Engineering and Physical Sciences, Heriot-Watt University, Edinburgh EH14 4AS, U.K.

## Abstract

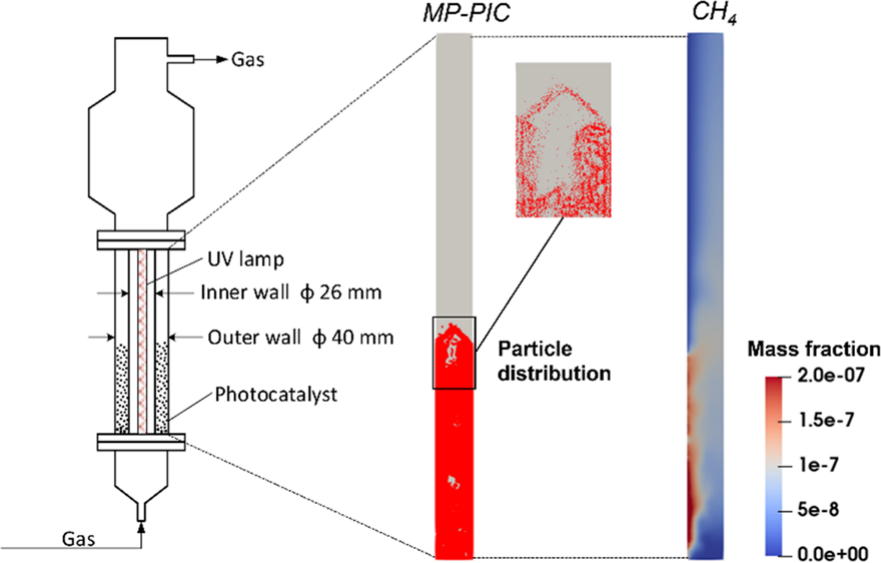

Carbon dioxide (CO_2_) photoreduction is a promising process for both mitigating CO_2_ emissions and providing chemicals and fuels. A gas–solid two-phase annular fluidized bed photoreactor (FBPR) would be preferred for this process due to its high mass-transfer rate and easy operation. However, CO_2_ photoreduction using the FBPR has not been widely researched to date. The Lagrangian multiphase particle-in-cell (MP-PIC) simulation with computational fluid dynamic models is a new and robust approach to explore the multiphase reaction system in the gas–solid fluidized bed. Therefore, the purpose of this paper is to investigate CO_2_ photoreduction in the FBPR by MP-PIC modeling to understand the intrinsic mechanism of solid flow, species mass transfer, and CO_2_ photoreaction. The MP-PIC models for solid flow in the FBPR were validated by the bed expansion height and bubble size. The results showed the particle stress of the Lun model, the drag of the Ergun-WenYu (Gidaspow) model, and the coefficient of restitution *e* = 0.95 with the wall parameters *e*_w_ = 0.9 and μ_w_ = 0.6 are the best fit to the experimental empirical correlations. The MP-PIC models developed in this work proved to be better than the Eulerian two-fluid modeling in the prediction of the bed expansion height and bubble size. It was also found from the simulation results that the maximum radiation intensity is in the half reactor height area, and the photocatalytic reaction mainly occurred around the inner wall. It showed that the gas velocity and catalyst loading were two crucial operating parameters to control the process. The results reported here can provide guidance for the operation and reactor design of the CO_2_ photoreduction process.

## 1 Introduction

CO_2_ photoreduction is attracting significant interest as it has the potential to decrease CO_2_ emissions as well as to provide chemicals and fuels.^1^ However, this process has a huge challenge in practical applications due to a very low conversion rate.

Previous research demonstrated that CO_2_ photoreduction is a mass-transfer-controlled process.^2,3^ Therefore, strategies to increase the mass-transfer rate are sought when using gas–liquid–solid three-phase photocatalytic reactors, such as a high CO_2_ pressure to increase CO_2_ solubility in the liquid;^4^ mechanical stirring; or gas bubbling.^5^ Additionally, some novel composite catalysts such as Cu_2_O nanoplatelets supported on multilayer graphene have been developed by Hurtado et al.^6^ for improving CO_2_ reduction due to the enhanced electron migration from the photocatalyst into the graphene. Ola and Maroto-Valer^7^ and Khan and Tahir^8^ summarized the photoreactors for CO_2_ photoreduction. They divided the photoreactors into three groups: (i) gas–liquid–solid three-phase slurry photoreactors, such as twin reactors; (ii) fixed-bed photoreactors, such as packed bed, thin-film bed, optical fiber bed, and monolith honeycomb bed reactors; and (iii) membrane photoreactors, such as slurry bed membrane and fixed-bed membrane reactors. Traditional gas–liquid–solid three-phase slurry photocatalytic reactors are not conducive to CO_2_ photoreduction because CO_2_ mass transfer has to take place in two difficult stages: from gas to liquid, where CO_2_ gas needs to be dissolved into the liquid and from liquid to the solid, where the soluble CO_2_ needs to be adsorbed on the surface of the photocatalyst. Disadvantages of the fixed bed photoreactors are the low mass-transfer rate, temperature gradient, the catalyst is difficult to replace, and the active life of the catalyst must be considered. Mozia^9^ presented that when the suspended photocatalysts are used, the efficiency of photocatalysis is usually much higher than that in the fixed-bed photoreactors, and the catalysts fixed on a carrier material, such as glass, quartz, wire mesh, or membrane, cause some shortcomings, such as low surface-to-volume ratios, less photocatalytically active surface, and inefficiencies introduced by the absorption and scattering of light through the reaction medium. The drawback of membrane photoreactors is a relatively low diffusion process, which is rate-determining, with membrane fouling, and a limited lifetime of the membrane.^9,10^

It is suggested that the annular photocatalytic gas–solid fluidized bed, which has been used for other photocatalytic processes,^11,12^ would be suitable for CO_2_ photoreduction because it presents the following intrinsic advantages: (i) CO_2_ is directly adsorbed onto the surface of the photocatalyst with a one-step mass-transfer process; (ii) highly efficient gas–solid contact; (iii) high mass- and heat-transfer rates; (iv) flexible operation, such as CO_2_ or photocatalyst recycling into the reactor column; and (v) convenient UV lamp arrangement. However, the application of the annular fluidized bed photoreactor (FBPR) for CO_2_ photoreduction is rarely reported.^13,14^

Computational fluid dynamic (CFD) modeling is a robust tool to numerically investigate multiphase flow and reactions. Specifically, CFD simulation relies on the experimental validation. Furthermore, it can also effectively analyze the physical mechanism and reaction behaviors to provide theoretical support for understanding the flow and reactions in the reactor. The successful simulation of gas–solid flow in a fluidized bed relies on solid particle modeling. Currently, there are two CFD methods, namely, Eulerian and Lagrangian modeling for researching the photocatalytic process. For Eulerian modeling, the solid phase is simplified and treated as the continuous phase. Thus, the kinetic theory of granular flow (KTGF) is introduced as the closure model to derive the solid pressure and solid viscosity. For example, Jing et al.^15^ simulated photocatalytic hydrogen production from the refinery gas of H_2_S in an annular fluidized bed by Eulerian gas–solid two-fluid modeling (TFM); Pareek et al.^16^ investigated pilot-scale annular bubble column photocatalytic reactors by the Eulerian gas–liquid–solid three-phase models. Generally, the Eulerian method is suitable for the simulation of the flow in large pilot-scale reactors with the number of particles being more than 10^9^.^17^ This method can save computing time and has low requirements for computer resources. The disadvantages of this method include the following: (i) solid particles are simplified as a continuous interpenetrating phase and averaged flow properties in each cell; (ii) the assumption of KTGF is only for perfectly smooth spheres, and only the normal impact and rebound velocities of colliding particles are taken into account neglecting the tangential impact; (iii) the particle velocity distribution is assumed only to match the Maxwell function equation; and (iv) it is not possible to trace individual particles or small-particle clusters in the computational domain.^18,19^ For Lagrangian modeling, Braham and Harris^20^ simulated the gas–solid flow in an annular fluidized bed photoreactor (FBPR) by discrete particle modeling (DPM). For DPM, the force between two particles is from elastic soft sphere collision, just like a spring. The dominated contact forces comprise the normal force and the tangential force. DPM is very close to the real physical particle collision process, but it is only suitable for simulation of the flow in small geometries because it is limited by the number of particles in the system, generally less than 10^6^ due to the computer memory size.^20^ In addition, DPM generally uses the explicit central difference time integration scheme to solve the particle motion, which is only conditionally stable with the stability determined by the size of the time-step.^21,22^ The required very small time-step, for example, 10^–6^ s, which is dependent on the particle stiffness and particle collision contact state, increases the computational time cost and makes the simulation expensive.

CFD Eulerian–Lagrangian multiphase particle-in-cell (MP-PIC) is a newly developed robust modeling method for solid flow, which adopts the concept of discrete particles or parcels in DPM. For this method, the motion of each particle or parcel follows the equation of Newton’s second law. The collision between particles is derived from the particle stress model. The MP-PIC method can achieve greater stability than other simulation methods by treating the solid particles as computational discrete particles or as a continuum phase. In this method, the particle properties are mapped from the Lagrangian coordinates to a Eulerian grid using interpolation functions, and after the evaluation of the continuum derivative terms, the particle properties are mapped back to the individual particles.^23^ The advantages of this method are as follows: (i) the MP-PIC for modeling particle collision is suitable for a dense solid flow without significant loss of accuracy;^19^ (ii) the MP-PIC method can be applied for the solid flow from small reactors including several hundreds of clouds with each cloud only consisting of one particle to industrial reactors using 10^6^ clouds/parcels to represent about 10^13^ particles;^24,25^ and (iii) the time step for the particle motion does not rely on the particle size because the particle interforce is modeled by the particle stress model without resolving individual particle collision using the averaged Eulerian field, so the MP-PIC modeling is significantly faster than DPM. Nowadays, this method has successfully simulated the heterogeneous gas chemistry of ozone decomposition and CO_2_ capture by dry amine-grafted sorbents in the fluidized bed with the particle motion predicted accurately.^26,27^ However, MP-PIC modeling has not been employed to numerically investigate the photocatalytic reactors. Therefore, in this work, we explore the use of the FBPR for CO_2_ photoreduction by investigating the flow behavior and reaction process with MP-PIC modeling. Accordingly, this paper focuses on this parcel-in-cell method to understand the physical behavior and reaction mechanism of CO_2_ photoreduction in the FBPR.

## 2 FBPR for the CO_2_ Photoreduction Process

**Figure 1:**
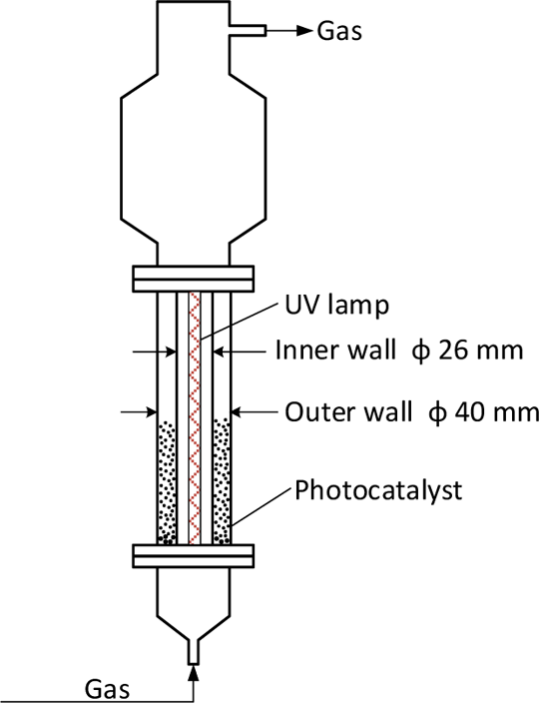
Schematic diagram for the FBPR.

A typical photocatalytic gas–solid fluidized bed is shown in Figure 1. For the annular column, the inner and outer diameters are 26 and 40 mm, respectively, as reported in ref (28). The gas distributor is made from a sintered glass plate. The photocatalyst is TiO_2_ coated on the particle of γ-Al_2_O_3_. The particle size is 107 μm and the particle density is 3246 kg/m^3^. The minimum fluidization velocity is 1.16 cm/s. An UV lamp is in the bed center. The details of the UV lamp are described in Section 3.2. The photocatalysts are packed in the annular column at the initial state before fluidization commences. The gas is composed of an inert carrier gas such as argon, and the reactant gases such as CO_2_ and water (H_2_O) moisture. The molar percentages of Ar, CO_2_, and H_2_O for the gas inlet are 72.36, 25.05, and 2.59%, respectively, which are calculated from the Ar, CO_2_, and H_2_O partial pressures.^29^ The H_2_O moisture was generated from a temperature-controlled saturator.^29^ This setting is following Thompson et al.’s work.^29^ The gas mixture is introduced into the column from the bottom at a constant velocity. Then, the photocatalyst particles are suspended fully if the gas velocity exceeds the minimum fluidization velocity and the bubbles flow from the bottom, rising through the bed and causing particles to be highly mixed and agitated. CO_2_ and H_2_O are adsorbed on the surface of TiO_2_ and activated to be reduced by the irradiation of UV light. Finally, the product gases, such as CH_4_, H_2_, and CO, and unreacted gases flow out of the column from the top.

## 3 Simulation Theory

In this work, the Lagrangian MP-PIC modeling was compared with Eulerian TFM. For MP-PIC modeling, the closure model is the Lun equation as the particle stress model with *e* = 0.95. The drag model is the Ergun-WenYu (Gidaspow) equation, and the isotropy model is applied if there is no special mention or explanation in the following sections. Here, the isotropy model is employed because we consider that the additional effect of particle collision drives the particle velocity distributions toward isotropy in the solid dense phase.^30^ For TFM, the closure model is the solid pressure using the equation from the Lun model and the solid viscosity using the equation from the Gidaspow model, and the drag model using the Ergun-WenYu (Gidaspow) equation. The detailed equations are presented below.

### 3.1 CFD Model

For Eulerian TFM, the Navier–Stokes equations were used, where the continuity equation is
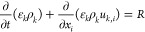
1where ε is the *k* phase volume fraction, dimensionless; ρ is the phase density, kg/m^3^; *u* is the velocity, m/s; *R* is the reaction mass source, kg/(m^3^·s); *t* is the time, s; and *x* is the *i*th coordinate direction, m.

The momentum equation is given below.
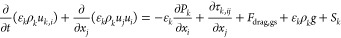
2where *P* is the pressure, Pa; *F*_drag,gs_ is the drag force between gas phase and particles, N; *S* is the momentum source, N/m^3^; *g* is the acceleration due to gravity, 9.81 m/s^2^; and τ is the stress tensor, Pa.

The species equation is
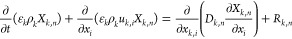
3where *X* is the mass fraction of *n* species, dimensionless and *D* is the diffusion coefficient, kg/(m·s).

For MP-PIC modeling, the equations for the gas phase are the same as the Eulerian models, but for the solid particles, the equations are^31,32^
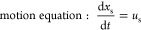
4
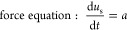
5
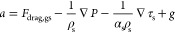
6where *a* is the acceleration, m/s^2^; *F*_drag,gs_ is the drag force between the gas phase and particles, N; *F*_drag,gs_ = *D*_gs_·(*u*_g_ – *u*_s_); *D*_gs_ is the drag coefficient, kg/(m^3^·s); and τ_s_ is the particle stress, Pa. The drag forces in eqs 2 and 6 are equal due to the momentum exchange between the gas phase and particles.

The particles in a cell are averaged by the dual-mesh Lagrangian procedure and a barycentric interpolation scheme is used.

### 3.2 Radiative Transport Equation

For UV light radiation, the radiative transport equation (RTE) was used to describe the light radiation intensity distribution in the bed. The RTE is
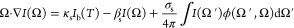
7where *I* is the radiative intensity at a given position following the Ω direction, W/m^2^; κ_s_, absorption coefficient, 1/m; β_s_, extinction coefficient, 1/m; and σ_s_, scattering coefficient, 1/m. Here, for TiO_2_ particles, the absorption coefficient is κ_s_ (1/m) = 3.598 × *W*_cat_ (g/m^3^) and the scattering coefficient is σ_s_ (1/m) = 0.2758 × *W*_cat_ (g/m^3^) and *W*_cat_ is the photocatalyst loading.^33^ The physical meaning of RTE (eq 7) is that the change of intensity is equal to the intensity enhancement due to emission, intensity reduction due to absorption and intensity enhancement due to scattering. *I*_b_ is the intensity from emission in the temperature field. The particle concentration influences the UV radiation through the absorption coefficient and scattering coefficient. During simulation, *W*_cat_ is equal to the solid density of the photocatalyst times solid fraction (ρ_s_ × ε_s_). Alvarado-Rolon et al.’s work on kinetic modeling of paracetamol degradation by photocatalysis considered the UV light absorptions at 254 nm by both the photocatalyst of TiO_2_ and the reagent of paracetamol.^34^ In this work, the UV absorption by CO_2_ is not considered because He et al.^35^ demonstrated that no absorption by CO_2_ is observed in the wavelength range of 307–725 nm. Moreover, the UV absorption by H_2_ is omitted in this work because the concentration of H_2_ in the gas is very low with the maximum mass fraction of about 10^–10^.

Generally, there are two methods for solving this equation. One is the discrete ordinate method (DOM), also known as the P–N method.^36−38^ The full angle can be divided into a series of number of discrete angular intervals and then resolve RTE along different angular directions. Another method is the P1, which is the simplest approximation of P–N models. It expands the RTE into a spherical isotropic model. Because the DOM method takes a long time and high computer resources, the P1 method is used in this work, which has been employed by other simulations.^39,40^

The intensity of radiation on the inner wall as the boundary condition can be described as the following equation.^41^
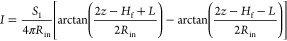
8where *R*_in_ is the radius of inner wall, m; *H*_f_ is the height of fluidized bed column, m; *L* is the height of the lamp, m; *z* is the size along the vertical direction, m; *S*_1_ is the UV lamp radiation function, *S*_1_ = 2π × *R*_lamp_ × *I*_lamp_; *R*_lamp_ is the radius of the lamp; and *I*_lamp_, radiation intensity from the lamp. In this work, *R*_in_ = 0.013 m; *H*_f_ = 0.1 m; *R*_lamp_ = 0.008 m; and *L* = 0.08 m.

**Figure 2:**
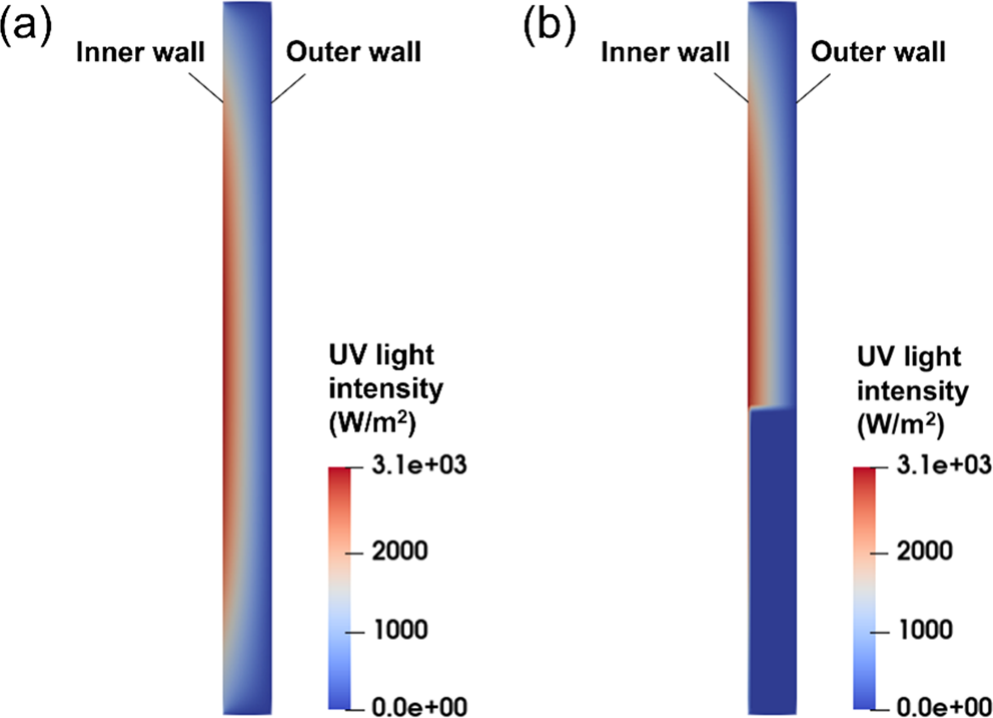
Radiation intensity distribution in the FBPR: (a) bed with rare tiny photocatalyst particles; (b) dense fluidized bed.

The light intensity from the UV lamp is set as 4000 W/m^2^ (400 mW/cm^2^) at a wavelength of 365 nm, which is the same as that in Thompson et al.’s work.^29^ The typical radiation intensity distributions with *I*_lamp_ = 4000 W/m^2^ are shown in Figure 2. The purpose of Figure 2 is to compare the radiation intensity distribution between a nearly empty column and a specific fluidized bed as an example. Figure 2a shows the distribution in the nearly empty bed with a very low solid fraction of 10^–10^ to imitate the rare tiny photocatalyst particles may float in the bed and Figure 2b shows the distribution in the dense fluidized bed with solid particles under the condition of *U*_g_ = 0.08 m/s and initial packed bed height, *H*_pack_ = 26 mm. In the nearly empty bed, the highest radiation intensity is in the bed center area. Along the vertical direction, the radiation intensity decreases along the direction from the bed center to the bed top and bed bottom. Along the horizontal direction, the radiation intensity decreases from the inner wall to the outer wall. These observations agree with the prior simulation and experimental work.^42^ In the fluidized bed with a bed expansion height of *H*_b_ = 40.2 mm and average solid fraction, ε_s_ = 0.26, the radiation intensity attenuates highly in the solid photocatalyst particles, where the attenuation degree is a function of the solid concentration. Therefore, it can be concluded that the CO_2_ photoreduction reaction mainly occurs in the thin area next to the inner wall.

### 3.3 Reaction Model

CO_2_ photoreduction is a complex reaction system, and the reaction mechanism and kinetics have been investigated.^43−46^ Generally, CO_2_ photoreduction is a mass-transfer-controlled process and thus the kinetic equation is widely accepted to be based on the Langmuir adsorption equation, rather than the Arrhenius rate equation.^44^ For the reaction model, the Thompson model based on the Langmuir equation is applied.^29^ Thompson et al.’s publication^29^ provided many informative data and parameters, which best fit to the simulation in this work and therefore, we employed parameters from Thompson et al.’s work. Furthermore, we are planning to carry further validation using cases and parameters from other publications and experiments. The Thompson model equation is the Weibull probability density function (PDF) multiplied with the Langmuir adsorption equation form as the reaction rate. In the Weibull PDF, the time and radiation intensity are introduced. Therefore, the reaction rate is the function of time, radiation intensity, and CO_2_ partial pressure and H_2_O partial pressure.

The reaction rates for generating CH_4_, CO and H_2_ are
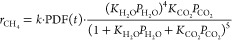
9
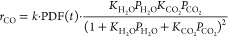
10
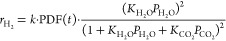
11where *r* is the reaction rate on the surface, μmol/(g_cat_·h); *k* is the rate constant, μmol/(g_cat_·h); PDF(*t*) is the Weibull PDF; *K* is the equilibrium adsorption constant, 1/bar; and *P* is the partial pressure, bar.

The Weibull PDF equation is
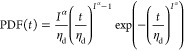
12where η_d_ is the deactivation scale parameter, dimensionless and α is the reaction order of light intensity, dimensionless. The Weibull PDF equation introduced by Thompson et al.^29^ considers that the active sites on the surface of the photocatalyst deactivate over time.

In this work, all the rection parameters for eqs 9–12 are picked up from ref (29). Generally, the CO_2_ photoreduction route is from CO_2_ to CO and finally to CH_4_. CO is a very important intermediate product and CH_4_ is a target product as the solar fuel. The mechanism of CO_2_ photoreduction to CH_4_ has been theoretically studied and demonstrated by Ji and Luo.^43^ H_2_ is produced from water splitting. Other products, such as formaldehyde and methanol, are the intermediate products and possible final products.^47,48^ It has been analyzed in the publication.^45^ From Thompson et al.’s experiments,^29^ CO, H_2_, and CH_4_ are the main final products and other publications have demonstrated it as well.^46^ Therefore, in this work, we consider CO, H_2_, and CH_4_ as the main final products by CO_2_ photoreduction.

The volumetric reaction rate, *r*_v,*i*_ in the modeling is
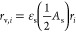
13

Here, we consider only the half particle area with a glazed side absorbs the UV light. *A*_s_ = *A*_ps_/*V*_p_. *A*_s_ is the solid surface area per volume, m^2^/m^3^; *A*_ps_ is the particle surface area, m^2^; and *V*_p_ is the particle volume, m^3^.

## 4 Simulation Method

The simulation studies were performed using OpenFOAM, which is a free and open-source toolbox providing different CFD robust solvers, such as multiphase flow and reaction solver, particle tracking solvers, and so forth, and providing the accessible and modifiable code interface. The assumptions used in this work are as follows: (i) the gas–solid flow in the annular column is isotropic and therefore, the 2D axisymmetric model and mesh are used in this work; (ii) the reactor is isothermal and therefore, the energy equation is not considered and applied in the simulation; (iii) the gas is incompressible with constant density and constant viscosity (gas density: ρ_g_ = 1.54 kg/m^3^; gas viscosity: μ_g_ = 2.64 × 10^–5^ Pa·s); (iv) the gas flow is in the laminar regime (Re_g_ = 30–100); (v) the gas velocity is uniform for the gas inlet area; (vi) the wall for gas flow is no slip wall; (vii) one parcel has only one particle and the particle size is a unique fixed value; and (viii) the CO_2_ photoreduction occurs after *t* = 0.5 s so that the particles are fully fluidized to prevent the impact of the flow transition from the fixed state to the fluidized state on the CO_2_ photoreduction reaction. Heat can be generated by the UV radiation, but one of the advantages of the fluidized bed is that very fast heat transfer occurs and thus, it can achieve a nearly uniform temperature distribution. Moreover, Thompson et al.^29^ reported no significant temperature rise caused by UV lighting. Therefore, in this work, we assumed that the temperature (314 K) is uniformly distributed in the bed. During simulation, we assume that all the boundary walls are transparent quartz walls and their influence on the UV irradiation is negligible. Thus, the boundary walls are set as the same as the internal field.

**Figure 3:**
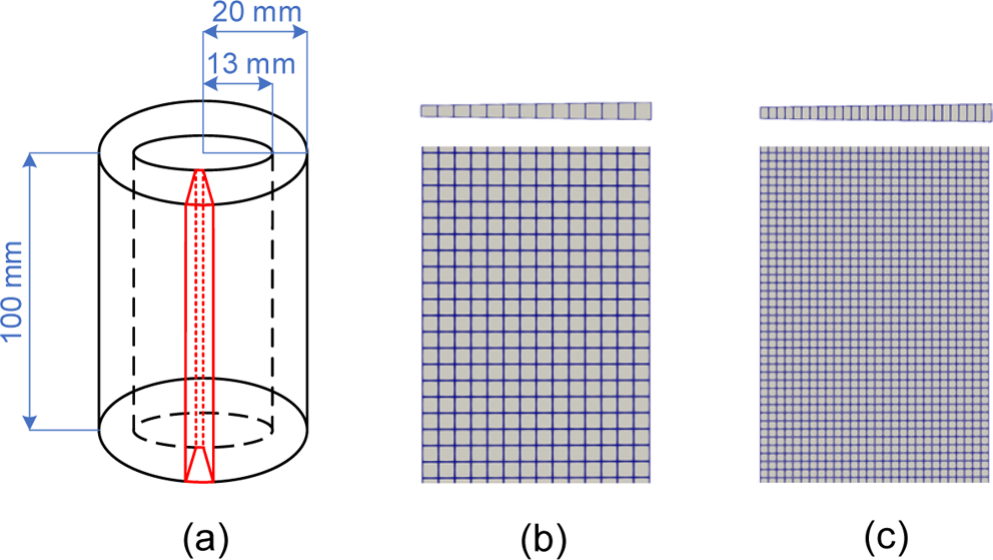
2D axisymmetric model and meshes: (a) 2D geometrical model; (b) mesh for Lagrangian MP-PIC modeling; (c) mesh for Eulerian TFM.

In this work, the 2D axisymmetric geometrical model used in the simulation is shown in Figure 3. The height is 100 mm, while the annular length is 7 mm, as shown in Figure 3a. The half single-layer angular angle is 0.8°. The mesh size should be more than one particle size, otherwise it may lose the physical meaning. The mesh for Lagrangian MP-PIC modeling is 14 × 200 and the size for each cell is 5 times of the particle diameter, *d*_p_, as shown in Figure 3b. The mesh for Eulerian TFM is 28 × 400, as shown in Figure 3c. The ratio of the mesh size to particle size is suggested from a prior published work.^49,50^

The discretization scheme is the Gauss limited linear. The time step is 10^–4^ s. The courant number, *C*, representing a particle stay in one cell of the mesh, *C* = *u*Δ*t*/Δ*h*, is 0.016 < 0.7 as the gas velocity is 0.08 m/s, which satisfies the convergence condition. Δ*t* is the simulation step time and Δ*h* is the mesh height. Each step simulation tolerance is less than 10^–5^. The numbers of particles are 78,390, 114,660, and 150,930 for the initial packed bed heights of 0.026, 0.038, and 0.050 m with a solid fraction of 0.6, respectively, which follows the rule of (*n*_particle_ × *V*_p_/*V*_packed bed_ = 0.6). *n*_particle_ is the number of particles; *V*_p_ is the particle volume; and *V*_packed bed_ is the volume of the initial packed bed. The maximum solid fraction for the packed bed is 0.63. It means that the local solid fraction in the dense flow would not exceed the maximum solid fraction and at this limitation, no particle collision occurs. The simulation time duration for evaluating the simulation parameters is 10 s. The simulation time duration for investigating operating parameters is 20 s.

## 5 Validation of Simulation

### 5.1 Validation Data

We validated the simulation using a bed expansion height, *H*, bubble size, *D*_b_, and mass fraction of CH_4_, *Y*_CH4_, based on a gas superficial velocity of 0.08 m/s. The experimental values of the bed expansion height and bubble size are derived from the empirical correlations from ref (28).

The average bed voidage is presented below
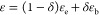
14where ε is the bed voidage, dimensionless; ε_e_ is the emulsion phase voidage, dimensionless; ε_b_ is the bubble phase voidage, dimensionless; and δ is the bubble phase fraction, dimensionless.

The bubble phase fraction is
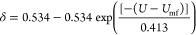
15where *U* is the gas superficial velocity, m/s and *U*_mf_ is the minimum fluidization velocity, m/s.

The emulsion phase voidage is given here
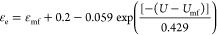
16

The bubble phase voidage is
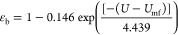
17

The bed expansion height is presented here
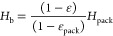
18where *H*_b_ is the bed expansion height, m; *H*_pack_ is the packed bed height, m; ε_pack_ = 0.6; and *H*_pack_ = 26 mm.

Due to the above correlations, it is derived that when the gas superficial velocity is 0.08 m/s and the bed expansion height is 41.1 mm.

The bubble generation and bubble rising in the bed are typical phenomena of gas–solid bubbling-fluidized beds. The bubble size, *D*_b_, can be estimated by the following correlation
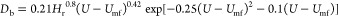
19

The bubble size, *D*_b_, is 4.1 mm when the gas superficial velocity is 0.08 m/s and the bed height, *H*_r_, the height between a point on the reactor bed column and the bed bottom, is 30 mm.

The mass fraction of CH_4_, *Y*_CH4_, is 10^–7^ to 10^–4^ through calculation from ref (29).

### 5.2 Effect of Particle Stress Models for MP-PIC Modeling

For MP-PIC modeling, the interaction force between particles is described by the particle stress. Therefore, the particle stress equation is crucial in accurately predicting the particle motion in the fluidized bed. In OpenFOAM, there are two models for describing the particle stress in the bed, which are the Lun model and Harris–Crighton model listed in the followings.

Lun model equation
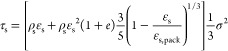
20where τ_s_ is the interparticle stress, Pa; *e* is the coefficient of restitution, dimensionless; ε_s,pack_ is the solid volume fraction of the packed bed, dimensionless; and σ is the root mean square (RMS) of velocity fluctuation.

Harris–Crighton model equation
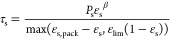
21where *p*_s_ and β are the coefficients for this model. Generally, *p*_s_ = 10 Pa and β = 2.

**Figure 4:**
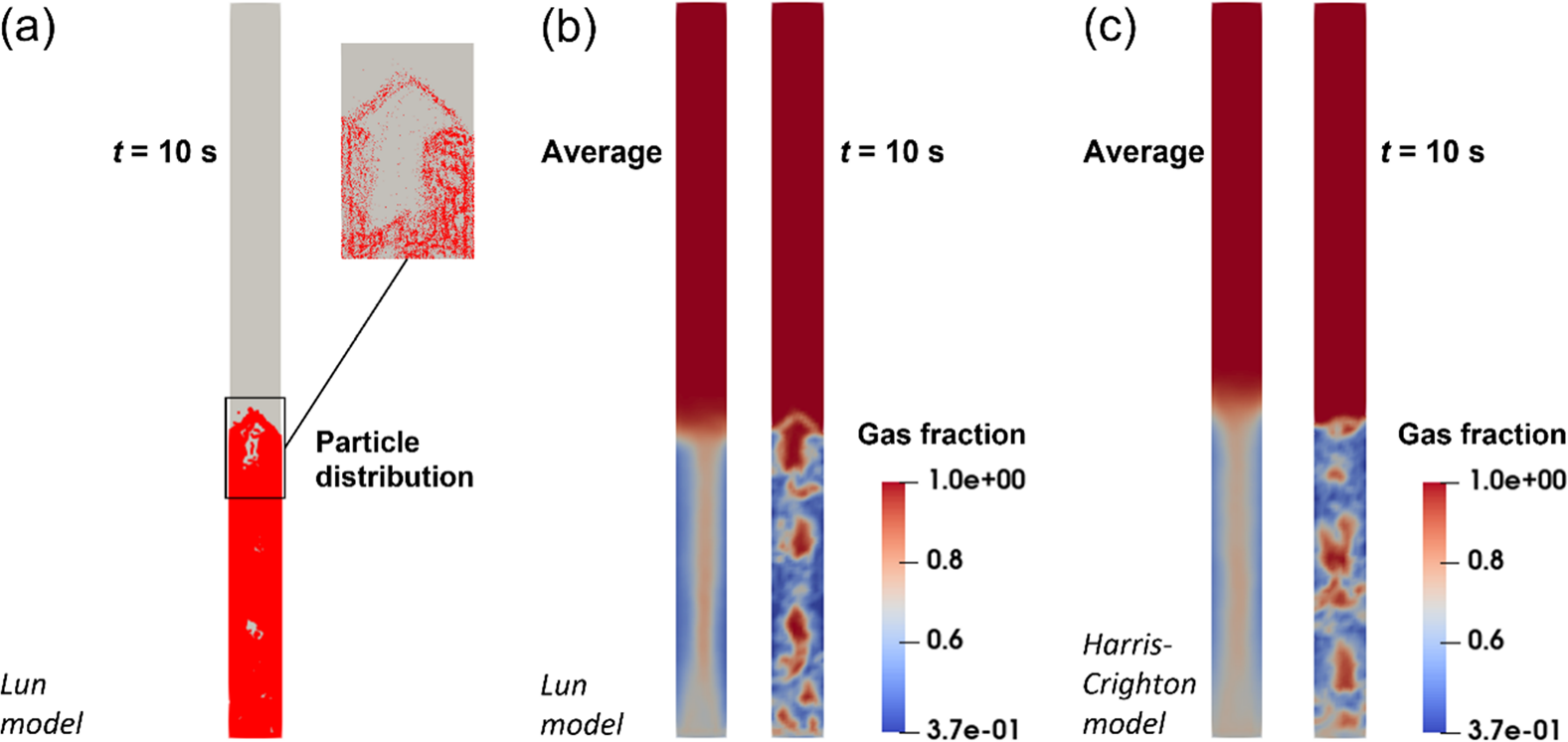
Comparison of particle stress models by Lagrangian MP-PIC modeling: (a) particle distribution with the Lun equation as the particle stress model; (b) gas fraction distribution with the Lun equation as the particle stress model; (c) gas fraction distribution with the Harris–Crighton equation as the particle stress model.

Figure 4 shows the particle distribution and gas fractions predicted by Lagrangian MP-PIC modeling with the Lun model and the Harris–Crighton model. In the simulation, *e* = 0.95 which is referred to refs (51–53), and the drag model is the Ergun-WenYu (Gidaspow) equation. The isotropy model is applied. Figure 4a shows the particle motion behaviors in the reactor predicted by MP-PIC modeling and where each particle motion can be traced. The purpose of Figure 4a is to show the prediction capability of MP-PIC modeling for particle cloud motion. Because of massive particle gathering, the solid fraction distribution cannot be identified clearly from this figure. However, the figures of the gas fraction distribution can indirectly display the solid fraction distribution. Therefore, Figure 4b,c for the gas fraction distribution were drawn and indirectly show the solid fraction distribution. The predicted bed height value is achieved from the averaged gas fraction distribution, as shown in the left picture of Figure 4b,c; thus in case to prevent the measurement error caused by the fluctuation of the bed surface. The bubble motion in the bed can be observed in the right picture of Figure 4b,c at *t* = 10 s. Finally, the corresponding bed expansion heights are 40.2 and 44.1 mm for the Lun model and Harris–Crighton model, respectively. It is found that MP-PIC modeling with the Lun model estimates the bed height very well with an error of 2.2%. The error of bed height predicted by MP-PIC modeling with the Harris–Crighton model is 7.3%, much higher than the real value. It can be concluded that the MP-PIC modeling with the Lun model is a suitable model for describing gas and solid flow in the FBPR through comparing the simulation results with experimental values of the bed expansion height from the correlation equation, eq 18.

### 5.3 Effect of Drag Models for MP-PIC Modeling

In order to investigate the drag models acting on the simulation of the FBPR, three drag models, namely, Ergun-WenYu (Gidaspow) model, Plessis–Masliyah model, and WenYu model were employed. The Ergun-WenYu drag model combines the Ergun equation when ε_g_ ≤ 0.8 and WenYu equation when ε_g_ > 0.8, as shown in eqs 22 and 23. The Plessis–Masliyah model is based on the Ergun equation of eq 22, but the coefficients *A* and *B* are estimated by eqs 24 and 25, respectively.

Ergun equation
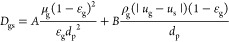
22where *D*_gs_ is the interphase momentum exchange coefficient, kg/(m^3^·s); *d*_p_ is the particle size, m; and *A* and *B* are coefficients, *A* = 150 and *B* = 1.75.

WenYu equation
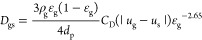
23where *C*_D_ is the drag coefficient.

Plessis–Masliyah equation
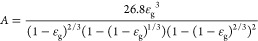
24
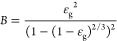
25

**Figure 5:**
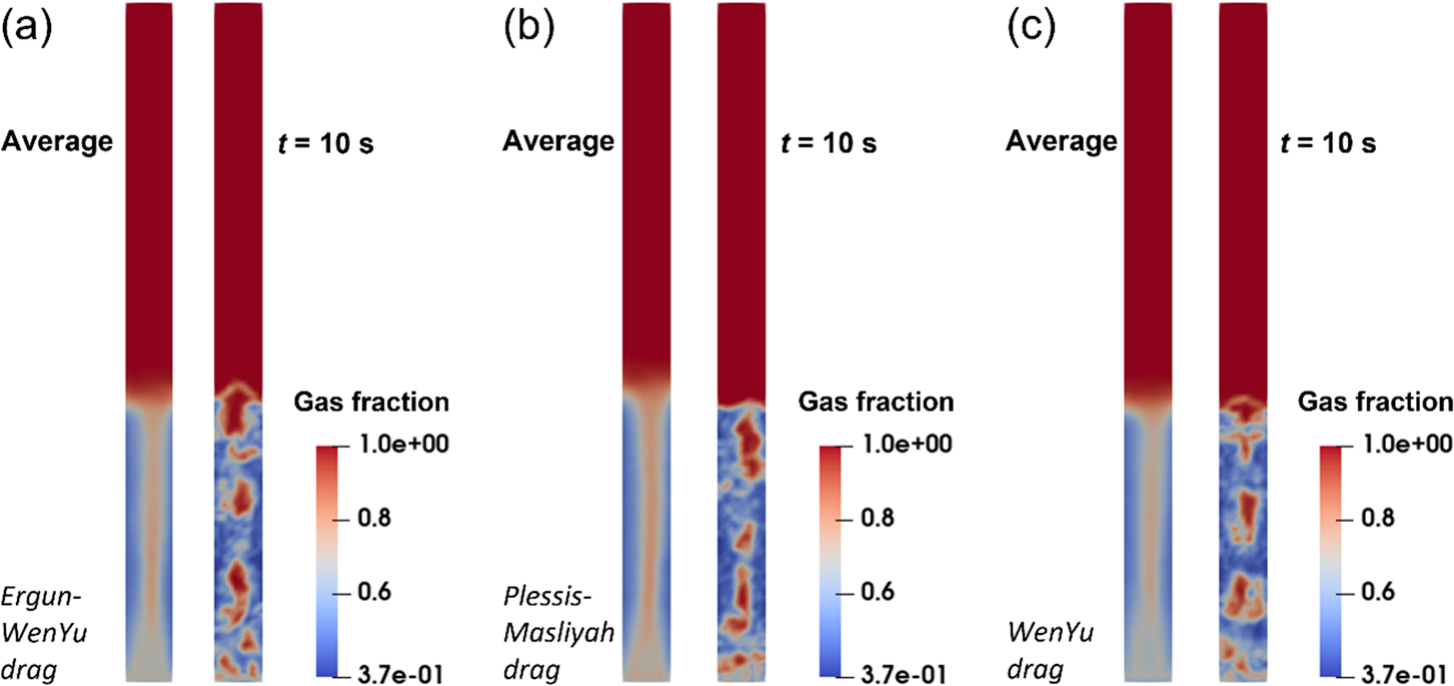
Effect of different drag models on the gas fraction distribution: (a) Ergun-WenYu (Gidaspow) drag model; (b) Plessis–Masliyah drag model; (c) WenYu drag model.

During simulation, the Lun model with *e* = 0.95 as the particle stress model and the isotropy model were applied. The simulation results of gas fraction distributions are shown in Figure 5, in which Figure 5a for the Ergun-WenYu drag, Figure 5b for the Plessis–Masliyah drag and Figure 5c for the WenYu drag. The corresponding estimated bed expansion heights are 40.2, 42.5, and 40.2 mm, respectively. In contrast to the experimental correlation value of bed height, 41.1 mm, the prediction error by the Ergun-WenYu drag and WenYu drag is only 2.2% but that by the Plessis–Masliyah drag is 3.4%. Additionally, the WenYu drag is generally suitable for the dilute phase with solid volume fractions below 0.2 and the Ergun-WenYu drag is suitable for solid volume fractions up to the packed state. Thus, it can be concluded that the Ergun-WenYu drag model is the best for predicting the particle flow in the annular FBPR because the predicted bed expansion height in this case is closest to the experimental value in the dense phase.

In a summary, the bed expansion height predicted by MP-PIC modeling is highly influenced by the particle stress models and drag models. It is demonstrated that the Lun particle stress model and the Ergun-WenYu drag model are suitable for describing the particle motion in the FBPR of this work.

### 5.4 Effect of Wall Boundary for MP-PIC Modeling

The interaction between the particles and the wall can be described by hard sphere collisions with the wall.^18^ The normal collision velocity and tangential velocity are presented as follows

For the normal direction
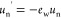
26where *e*_w_ is the coefficient of restitution for the particle and wall, dimensionless and *u*_*n*_ is the velocity with the normal direction of the wall, m/s.

For the tangential direction
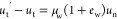
27where *u*_t_ is the velocity with the tangential direction to the wall, m/s and μ_w_ is the coefficient of friction for the wall, dimensionless.

**Figure 6:**
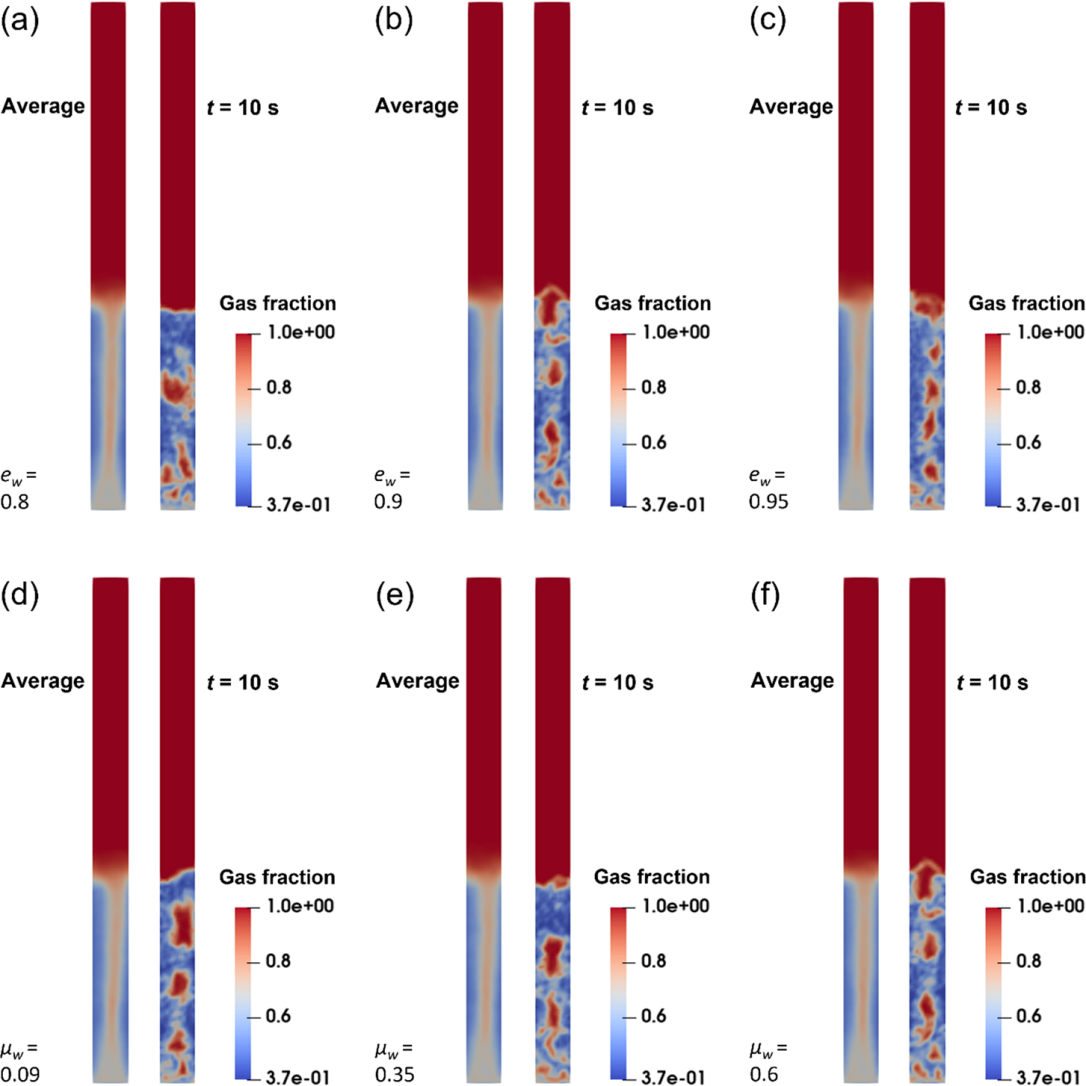
Effect of wall function parameters on the gas fraction distribution. Coefficient of restitution (μ_w_ = 0.6): (a) *e*_w_ = 0.8, (b) *e*_w_ = 0.9, and (c) *e*_w_ = 0.95; coefficient of friction (*e*_w_ = 0.9): (d) μ_w_ = 0.09, (e) μ_w_ = 0.35, and (f) μ_w_ = 0.6.

The coefficient of restitution for collision between the particles and wall, *e*_w_, and the coefficient of friction between particles and wall, μ_w_, are key parameters, thus the effects of these parameters on the flow behaviors in the bed have been studied. The results of gas fractions with different *e*_w_ and μ_w_ are shown in Figure 6. Figure 6a–c are shown under different *e*_w_ with the same μ_w_ of 0.6. Figure 6d–f are shown under different μ_w_ with the same *e*_w_ of 0.9. It indicates that all the bed expansion heights are the same, as 40.2 mm, but the bubble sizes are different. It is discovered that the wall boundary does not affect the bed expansion height but influences the bubble size. In this work, the bubble size is determined by averaging the bubble vertical size and horizontal size.

It can be seen from Figure 6a–c that when *e*_w_ increases from 0.8 to 0.95, the bubble size decreases from 4.6 to 4.3 and 3.5 mm, respectively. It demonstrates that *e*_w_ = 0.9 is the best for predicting the particle flow dynamics in the FBPR with the lowest prediction error. Figure 6d–f shows the particle flow under different μ_w_ = 0.6, 0.35, and 0.09. With the wall coefficient of friction decreasing, the bubble size increasing from 4.3 to 5.2 mm. As μ_w_ = 0.6, the predicted bed expansion height matches the experimental value with the lowest error of 2.2%.

It concluded that the wall function parameters affect the bubble size heavily. This is because the ratio of the bubble size to the narrow annular size is 4/7. Therefore, the wall zone influences the bubble formation and bubble coalescence significantly. Due to the bubble motion and size, it is concluded that *e*_w_ = 0.9 and μ_w_ = 0.6 are the optimum values for describing the particle flow in the studied FBPR because the predicted bubble size in this case is closest to the experimental value.

### 5.5 Comparison between TFM and MP-PIC

**Figure 7:**
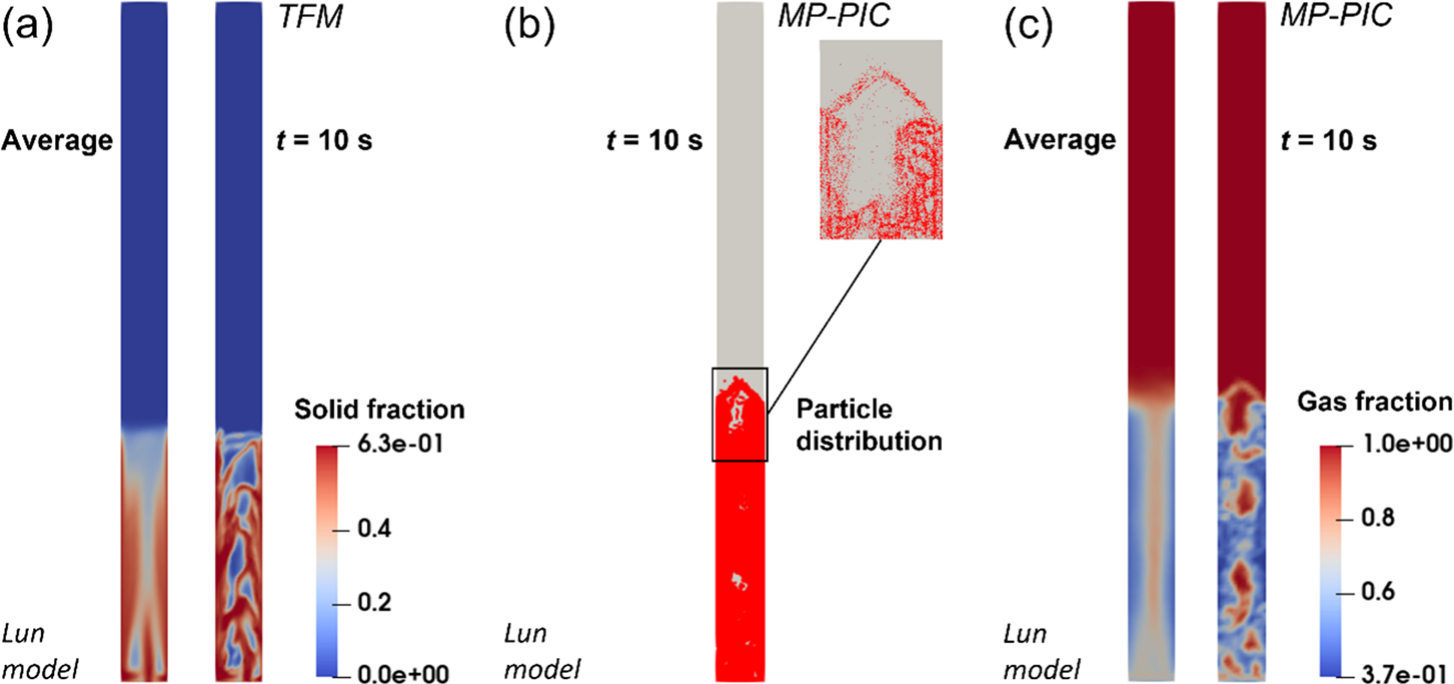
Comparison of simulations with Eulerian TFM and Lagrangian MP-PIC modeling: (a) solid fraction distribution predicted by TFM; (b) particle distribution predicted by MP-PIC modeling; (c) gas fraction distribution predicted by MP-PIC modeling.

It is well known that the particle flow dynamics in the bubbling fluidized bed can be efficiently simulated by TFM as well. For comparing the effectiveness of TFM and MP-PIC modeling on solid flow in the FBPR, the simulation by TFM was performed with the Ergun-WenYu drag model, *e* = 0.95 and the particle pressure using the Lun equation, and these conditions and models are the same with those by MP-PIC modeling. The comparison between TFM and MP-PIC modeling is shown in Figure 7. Figure 7a shows the solid distribution predicted by TFM. Figure 7b shows the particle distribution predicted by MP-PIC modeling and for observing the bubble clearly, the gas fraction estimated by MP-PIC modeling is shown in Figure 7c. The bed expansion heights predicted by TFM and MP-PIC modeling are 37.0 and 40.2 mm, respectively, with prediction errors of 10.0% and 2.2%, respectively. The bubble sizes predicted by TFM and MP-PIC modeling are 3.3 and 4.3 mm, respectively, with prediction errors of 19.5 and 4.9%, respectively. The bed expansion height and the bubble size estimated by TFM are much smaller than the real values. It expresses that MP-PIC modeling on the FBPR is better than TFM.

In addition, the differences of particle distributions simulated by TFM and MP-PIC modeling are on the locations of the bed bottom and bed top, as shown in the average solid fraction for TFM and average gas fraction for MP-PIC modeling. The MP-PIC model predicts that the bottom of the bed is a trapezoidal air cavity, while TFM predicts that the trapezoidal air cavity at the bottom of the bed is divided into two parts by a particle strip. The MP-PIC model predicts that the top of the bed is in the shape of a shallow plate and the side wall is a high-concentration area of particles, while TFM predicts that the top of the bed is in the shape of a goblet. The specific particle distribution at the top and bottom of the bed needs to be further verified by experimental imaging technology.

In order to further inspect the parameter settings on MP-PIC modeling, the bed expansion heights at the gas superficial velocities of 0.08, 0.16, and 0.24 m/s were used to validate the MP-PIC simulation by the empirical correlation equations. From the correlation equations, the bed expansion heights are 41.1, 44.4, and 47.4 mm, respectively, and the corresponding expansion height predicted by MP-PIC modeling are 40.2, 43.3, and 48.8 mm at the velocities of 0.08, 0.16, and 0.24 m/s, respectively. The prediction errors are 2.2, 2.5, and 2.9%, respectively. These results further prove that the current settings for MP-PIC models are the best fit to the experiment.

### 5.6 CO_2_ Photoreduction Process

**Figure 8:**
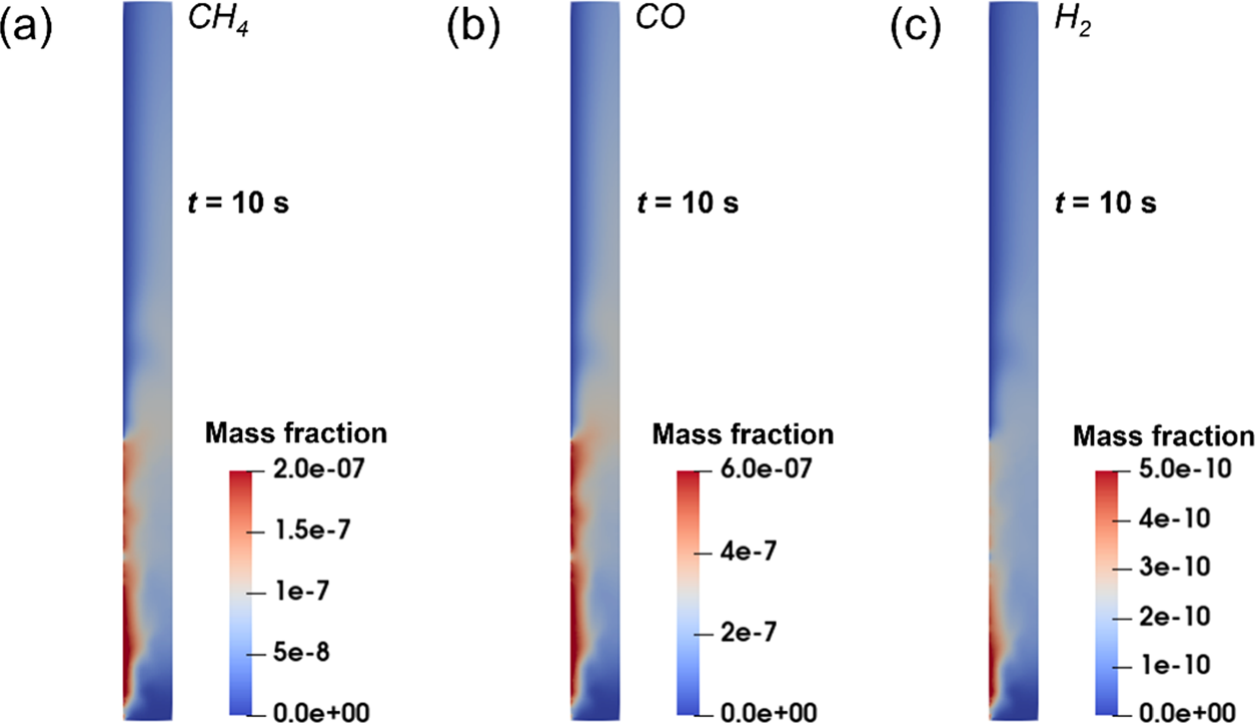
Production of CH_4_, CO, and H_2_ through CO_2_ photoreduction predicted by MP-PIC modeling: (a) CH_4_, (b) CO, and (c) H_2_.

The produced CH_4_, CO, and H_2_ mass fractions from CO_2_ photoreduction predicted by MP-PIC modeling using the Ergun-WenYu drag model under *e*_w_ = 0.9 and μ_w_ = 0.6 are shown in Figure 8. The gas velocity is 0.08 m/s. It can be seen that CO_2_ photoreduction occurs mainly in the area near the inner wall because it receives UV light. Figure 8 also shows that the local reaction intensity and product concentration have a close relationship with the local UV light intensity and solid photocatalyst concentration. The maximum CH_4_, CO, and H_2_ mass fractions are 3.1 × 10^–7^, 8.5 × 10^–7^, and 7.6 × 10^–10^, respectively. The maximum CH_4_ mass fraction is in the range of the experimental values reported^29^ and therefore, it validates the reaction model and parameter settings used in this work.

## 6 Simulation Results and Discussion for Operation Parameters

In this section, the simulations were performed under the following conditions: Ergun-WenYu (Gidaspow) drag model, Lun particle stress model with *e* = 0.95, wall coefficient of restitution with *e*_w_ = 0.9, and wall coefficient of friction with μ_w_ = 0.6.

### 6.1 Effect of Gas Velocity on Solid Flow and CO_2_ Photoreduction

**Figure 9:**
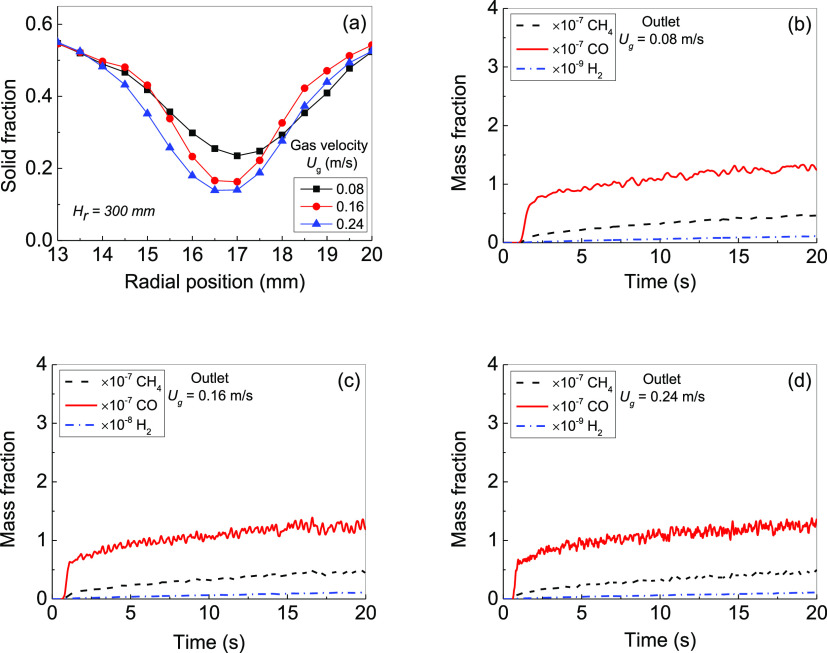
Effect of gas velocity on CO_2_ photoreduction: (a) solid fraction distribution along the radial direction at *H*_r_ = 30 mm under different gas velocities; (b–d) generated CH_4_, CO, and H_2_ mass fractions with time at the outlet for different gas velocities of (b) 0.08, (c) 0.16, and (d) 0.24 m/s.

Regulating gas velocity is commonly used for controlling solid flow and CO_2_ photoreduction. The simulations here were carried out with gas velocities of 0.08, 0.16, and 0.24 m/s. As the gas velocity rises, the bed expansion height increases significantly from 40.2 to 43.3 and 48.0 mm with the bubbles going through the bed center. With the gas velocity increasing, the bubbles coalesce forming a long-crooked strip in the bed center area. In order to investigate the solid fraction distribution along the radial position, the simulation data were averaged from *t* = 0.5 s to *t* = 20 s. The averaged solid fraction distributions at bed height *H*_r_ = 30 mm are shown in Figure 9a. It shows that the solid concentration near the wall around ε_s_ = 0.54 is higher than that in the bed center (ε_s_ = 0.1–0.3). Weber and Mei^54^ demonstrated that the solid fraction near the wall is around 0.5–0.6 and the solid fraction in the bed center is about 0.3 when *U*_g_/*U*_min_ = 6. This demonstrated that our current simulation with *U*_g_/*U*_min_ = 6.9 when *U*_g_ = 0.08 m/s is reasonable, as the solid concentration in the bed center (ε_s_ = 0.1–0.3) and near the wall (ε_s_ = 0.54), which is in coincidence with the experimental results in ref (54) with similar conditions. It further indicates that the settings of simulation parameters in the MP-PIC model of this work are fitted with predicting the particle flow in the reactor. The solid concentration near the inner wall is slightly higher than that near the outer wall. For example, when the gas velocity is 0.08 m/s and ε_s_ are 0.55 and 0.51 for the inner wall and outer wall, respectively. This may be caused by the axisymmetric annular structure with a small inner wall area and large outer wall area. This phenomenon was also demonstrated by Khan and Shamim.^55^

The influence of gas velocity on CO_2_ photoreduction relies on the solid photocatalyst distribution, especially near the inner wall and therefore, high gas velocity may increase CO_2_ photoreduction. With gas velocity increasing, the enhanced bed expansion enlarges the solid phase area receiving UV light. However, this also decreases the solid concentration near the wall and may reduce CO_2_ photoreduction. Therefore, the CH_4_ yield from CO_2_ photoreduction is a function of local radiation intensity and local photocatalyst concentration. This is due to the radiation intensity not being distributed uniformly, with the highest value in the middle of the reactor height and lowest in the top side and bottom side of the reactor. The solid photocatalyst concentration distribution is strongly determined and can be controlled by the gas velocity. Therefore, it seems that CO_2_ photocatalysis can be optimized by the gas velocity to change the photocatalyst concentration distribution along the bed height.

The variations of CH_4_, CO, and H_2_ mass fractions at the gas outlet with time are shown in Figure 9b–d. When the time started from *t* = 0.5 s, the CH_4_, CO, and H_2_ mass fractions increase until *t* = 20 s. However, the simulation results indicate that the mass fractions of CH_4_, CO, and H_2_ at the gas outlet are affected by the gas velocity very slightly. For example, at *t* = 20 s, CH_4_ mass fractions at the gas outlet for *U*_g_ = 0.08, 0.16, and 0.24 m/s are 0.49 × 10^–7^, 0.44 × 10^–7^, and 0.48 × 10^–7^, respectively; CO mass fractions at the gas outlet are 1.35 × 10^–7^, 1.19 × 10^–7^, and 1.32 × 10^–7^, respectively; H_2_ mass fractions at the gas outlet are 1.16 × 10^–10^, 1.06 × 10^–10^, and 1.12 × 10^–10^, respectively. The possible reason for this situation is that the initial packed bed height remains unchanged. As the gas velocity increases, the bed expansion height increases with enhancing the possibility of receiving UV light, but the corresponding particle concentration decreases locally and the total photocatalyst surface area correspondingly decreases for receiving light, resulting in the concentration of CH_4_ generated by CO_2_ photoreduction at the outlet basically unchanged with above different gas velocities. Furthermore, the oscillation of the mass fraction is intensified by higher gas velocity. The oscillation of the mass fraction, as shown in Figure 9b–d, is caused by the variation of the solid concentration and pressure under the gas flow, which resulted from the nonlinear dynamic motion of heterogeneous flow structures in gas–solid fluidization. Cui et al.^56^ demonstrated that with increasing gas velocity, the oscillation frequency raises, and the amplitude enlarges.

For further demonstrating the effect of gas velocity on CO_2_ photoreduction, the simulations were carried out based on the initial packed bed height of 38 mm with *U*_g_ = 0.08, 0.16 and 0.24 m/s. For different *U*_g_, the corresponding CH_4_ mass fraction is 0.72 × 10^–7^, 0.66 × 10^–7^ and 0.66 × 10^–7^, respectively. Similarly, the corresponding CO mass fraction is 2.09 × 10^–7^, 1.84 × 10^–7^ and 1.85 × 10^–7^, respectively. The H_2_ mass fraction is 1.59 × 10^–10^, 1.50 × 10^–10^, and 1.52 × 10^–10^, respectively. This further indicates that the gas velocity influences the product yield very slightly. Only at a low velocity of 0.08 m/s, the CH_4_ yield is slightly higher (8%) than that at higher gas velocity.

In brief, CH_4_ generated by CO_2_ photoreduction in the FBPR indicates that CO_2_ photoreduction process mainly occurs near the inner wall. The gas velocity influences the generation of CH_4_, CO, and H_2_ very slightly.

### 6.2 Effect of Initial Packed Bed Height on Flow and CO_2_ Photoreduction

The packed bed height is the height between the top and the bottom of the filled photocatalysts in the reactor column at *U*_g_ = 0 m/s, which physically means the photocatalyst loading in the bed. Catalyst loading can affect CO_2_ photoreduction by: (1) increasing photocatalyst concentration in the bed and (2) increasing the area of the solid phase exposing to the UV light. The simulations were performed with the initial packed bed heights of 26, 38, and 50 mm.

**Figure 10:**
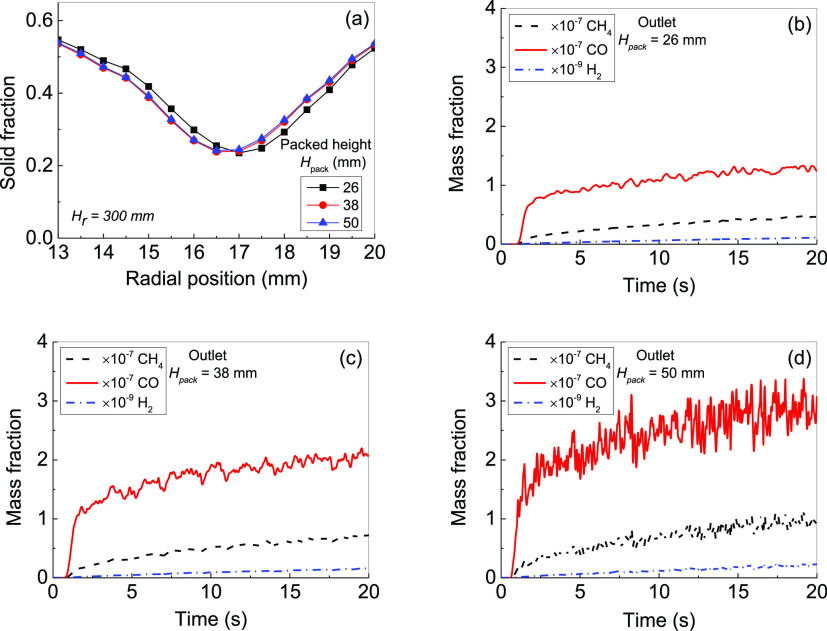
Effect of the initial packed bed height on CO_2_ photoreduction: (a) solid fraction distribution along the radial direction at *H*_r_ = 30 mm with different initial packed heights; (b–d) generated CH_4_, CO, and H_2_ mass fractions with time at the outlet for different initial packed bed heights of (b) 26, (c) 38, and (d) 50 mm.

The simulation results display that the bed expansion height increases sharply from 40.2 to 64.6 and 82.7 mm with the initial bed height increasing from 26 to 38 and 50 mm under the same gas velocity, *U*_g_ = 0.08 m/s. The bed expansion height is over the half bed height which is the position with the maximum radiation intensity. Figure 10 shows the simulation results for the solid flow and photoreaction. The solid fraction distributions at the bed height *H*_r_ = 30 mm are shown in Figure 10a. Although the initial packed bed height rises from 26 to 50 mm, the solid fraction distribution at *H*_r_ = 30 mm is nearly the same with ε_s_ ≈ 0.54 near the wall and ε_s_ ≈ 0.24 in the bed center.

Figure 10b–d shows the variation with the time of mass fractions of CH_4_, CO, and H_2_ at the gas outlet. It can be seen clearly that the CH_4_ mass fraction obviously increases with the initial packed bed heightened. For example, under *U*_g_ = 0.08 m/s and different initial packed heights of 26, 38, and 50 mm, the CH_4_ mass fractions at the gas outlet are 0.49 × 10^–7^, 0.72 × 10^–7^, and 1.02 × 10^–7^, respectively; the CO mass fractions at the gas outlet are 1.35 × 10^–7^, 2.09 × 10^–7^, and 2.96 × 10^–7^, respectively. The H_2_ mass fractions at the gas outlet are 1.16 × 10^–10^, 1.59 × 10^–10^, and 2.21 × 10^–10^, respectively. This indicates that the higher initial packed bed height, the higher product yield.

It can be concluded that increasing the initial packed bed height or photocatalyst loading enhances CO_2_ photoreduction to generate more CH_4_ in the FBPR. This is due to (i) the increase of the photocatalyst loading extending the surface area for CO_2_ adsorption and photoreaction and (ii) the increase of the bed height leading to more particles receiving the highest intensity of UV light because the radiation intensity is not distributed uniformly with the highest value in the middle of the reactor height.

## 7 Conclusions

This work investigated solid photocatalyst flow and CO_2_ photoreduction in a FBPR by CFD Lagrangian MP-PIC modeling. The simulation on the hydrodynamics of particle flow in the fluidized bed has been validated by the empirical correlations. These empirical correlations for predicting the particle flow and the reaction kinetic equations have been demonstrated to be effective, as shown in refs (28) and (29), respectively. In this work, in addition to validating the simulation by empirical correlations or experiments, we also demonstrated that CFD can be used to analyze the flow and reaction behaviors. The results reported here can provide guidance for the reactor design and operation of CO_2_ photoreduction using the FBPR.

First, different particle stress models, drag models, and simulation parameters, such as coefficient of restitution, *e*_w_, and coefficient of friction, μ_w_, have been studied and evaluated for the simulation of particle flow in the FBPR. The results show that the Lun particle stress model with *e* = 0.95 and the Ergun-WenYu (Gidaspow) drag model with *e*_w_ = 0.9 and μ_w_ = 0.6 are the best selection to describe the solid flow, particle interforce, and particle intercollision, which is validated by the experimental values of the bed expansion height and bubble size from the empirical correlation equations. It also indicates the model selections discovered and matched the intrinsic mechanism and rules of photocatalyst flow in the annular fluidized bed. Moreover, the Lagrangian MP-PIC modeling is compared with the Eulerian TFM in this work. It is concluded that the MP-PIC method is better than the TFM method in the field of the bed expansion height and bubble size. TMF treats the solid flow as a continuous phase using an uniform average flow characteristic under each cell and ignores the heterogeneity of particle clustering in a cell. This may cause the distortion of the simulation results. This has been discussed in detail in ref (57). The MP-PIC model treats the particles as the discrete sphere clouds and considers the difference of particle movement in a cell. Therefore, MP-PIC modeling is advantageous over TFM with considering a heterogeneous particle motion under one cell. The CH_4_ mass fraction by CO_2_ photoreduction was validated by Thompson et al.’s experiments.

Furthermore, the effects of operating parameters, such as the gas velocity and initial packed bed height on CO_2_ photoreduction in the FBPR, have also been investigated. The variation of the gas velocity influences the production of CH_4_, CO, and H_2_ very slightly, but the oscillation of the mass fraction increases with the gas velocity. The mass fraction of CH_4_, CO, and H_2_ increases at the gas outlet when the solid catalyst load is increased.

It can be seen that CO_2_ photoreduction mainly occurred in the area next to the inner wall. The maximum radiation intensity is located on the inner wall at the half of the bed height. This suggests that the solid catalyst loading should be more than half of the reactor column height. In addition, the reactor design and operating parameters for increasing the solid concentration on the inner wall can be optimized for improving CH_4_ productivity due to the increase of the photocatalyst particle concentration on the wall and enlargement of UV light adsorption and reaction surface area.

The CO_2_ photoreduction in the annular FBPR is a complex system, which is influenced by many factors and parameters from material physical properties, reactor dimensions, UV lighting, as well as operation conditions and reaction kinetics. Nevertheless, this work has successfully demonstrated that theoretical CFD models in combination with the UV radiation model and reaction kinetic model can help us to unravel the physical and reaction mechanisms of the CO_2_ photoreduction process.

The MP-PIC modeling developed here is an effective robust tool not only to understand the physical behavior of photocatalysts but also to explore the reaction mechanism of CO_2_ photoreduction process in the FBPR. The simulation method and results reported here can help optimize the design and operation of the FBPR, where the following factors should be considered: (i) the catalyst loading or initial packed bed height enhances the CO_2_ photoreduction to CH_4_; (ii) the gas velocity only increases the CH_4_ generation oscillation; (iii) the internal wall structures, such as the internal ring, are set up to increase the photocatalyst concentration near the wall and then enhance the CO_2_ photoreduction; and (iv) the optimum arrangement of the UV lamp around the inner and outer walls enhances the UV absorption by the photocatalyst.

## Nomenclature

*A*_s_: solid surface area per volume (m^–1^)
*a*: acceleration (m s^–2^)
*C*: courant number (—)
*C*_D_: drag coefficient (—)
*D*: diffusion coefficient (kg m^–1^ s^–1^)
*D*_b_: bubble size (m)
*D*_gs_: interphase momentum exchange coefficient (kg m^–3^ s^–1^)
*d*_p_: particle size (m)
*e*: coefficient of restitution (—)
*e*_w_: coefficient of restitution for wall (—)
*F*_drag,gs_: drag force (N)
*g*: acceleration due to gravity (m s^–2^)
*H*_b_: bed expansion height (m)
*H*_f_: height of the fluidized bed column (m)
*H*_pack_: initial packed bed height (m)
*H*_r_: height between a point on the reactor bed column and the bed bottom (m)
*h*: height (m)
*I*: radiative intensity (W m^–2^)
*I*_lamp_: radiation intensity from the lamp (W m^–2^)
*k*: reaction rate constant (μmol g_cat_^–1^ h^–1^)
*K*: equilibrium adsorption constant (bar^–1^)
*L*: height of the lamp (m)
*n*_particle_: number of particles (—)
*P*: pressure (Pa or bar)
*p*_s_: coefficient in eq 20 (—)
PDF(*t*): Weibull probability density function
*r*: reaction rate (μmol g_cat_^–1^ h^–1^)
*r*_v_: volumetric reaction rate (kg m^–3^ s^–1^)
*R*: reaction mass source (kg m^–3^ s^–1^)
*R*_in_: radius of the inner wall (m)
*R*_lamp_: radius of the lamp (m)
*S*: momentum source (N m^–3^)
*S*_1_: UV lamp radiation function (—)
*t*: time (s)
*u*: velocity (m s^–1^)
*u*_n_: velocity with the normal direction of the wall (m s^–1^)
*u*_t_: velocity with the tangential direction to the wall (m s^–1^)
*U*: gas superficial velocity (m s^–1^)
*U*_mf_: minimum fluidization velocity (m s^–1^)
*V*_packed bed_: volume of the packed bed (m^3^)
*V*_particle_: one particle volume (m^3^)
*W*_cat_: photocatalyst loading (g m^–3^)
*x*: ith coordinate direction (m)
*X*: mass fraction of the species (—)
*z*: size along the vertical direction (m)

## Greek

α: reaction order of light intensity (—)
β_s_: extinction coefficient (m^–1^)
δ: bubble phase fraction (—)
ε: phase volume fraction or bed voidage (—)
ε_b_: bubble phase voidage (—)
ε_e_: emulsion phase voidage (—)
ε_s,oacked_: solid volume fraction for the packed bed (—)
η_d_: deactivation scale parameter (—)
κ_s_: absorption coefficient (m^–1^)
μ_w_: coefficient of the friction on the wall (—)
ρ: phase density (kg m^–3^)
σ: root mean square of velocity fluctuation (—)
σ_s_: scattering coefficient (m^–1^)
τ: stress tensor (Pa)
τ_s_: particle normal stress (Pa)
Ω: direction (—)

## Subscript

*i, j*: *i*th or *j*th coordinate
*k*: *k*th phase, gas, or solid
*n*: *n*th species
CH_4_: methane
CO: carbon monoxide
H_2_: hydrogen
H_2_O: water
